# Supplementary material for: Low-dose ethanol consumption inhibits neutrophil extracellular traps formation to alleviate rheumatoid arthritis
Source: Commun Biol. 2023 Oct 26;6:1088. doi: 10.1038/s42003-023-05473-y (PMC10603044; doi:10.1038/s42003-023-05473-y)
Supplement: Supplementary file 2 — Supplementary Information [file 42003_2023_5473_MOESM2_ESM.pdf]

Supplementary Information

Low-dose ethanol consumption inhibits neutrophil extracellular traps formation to alleviate rheumatoid arthritis

Lin Jin[123#], Ziwei Zhang[123#], Pin Pan[4#], Yuchen Zhao[123], Mengqi Zhou[123], Lianghu Liu[123], Yuanfang Zhai[123], Han Wang[123], Li Xu[123], Dan Mei[123], Han Zhang[123], Yi'ning Yang[123], Jinghan Hua[123], Xianzheng Zhang[123*], Lingling Zhang[123*]

***Correspondence:**
Dr. Xianzheng ZHANG and Prof. Ling-ling ZHANG
E-mail address: zxzhang0514@163.com (X.Z. ZHANG);
ll-zhang@hotmail.com (L.L. ZHANG).
Phone: +86-0551-6516-1206; Fax: +86-0551-6516-1208

Supplementary Figures and Videos

1 Supplementary Figures

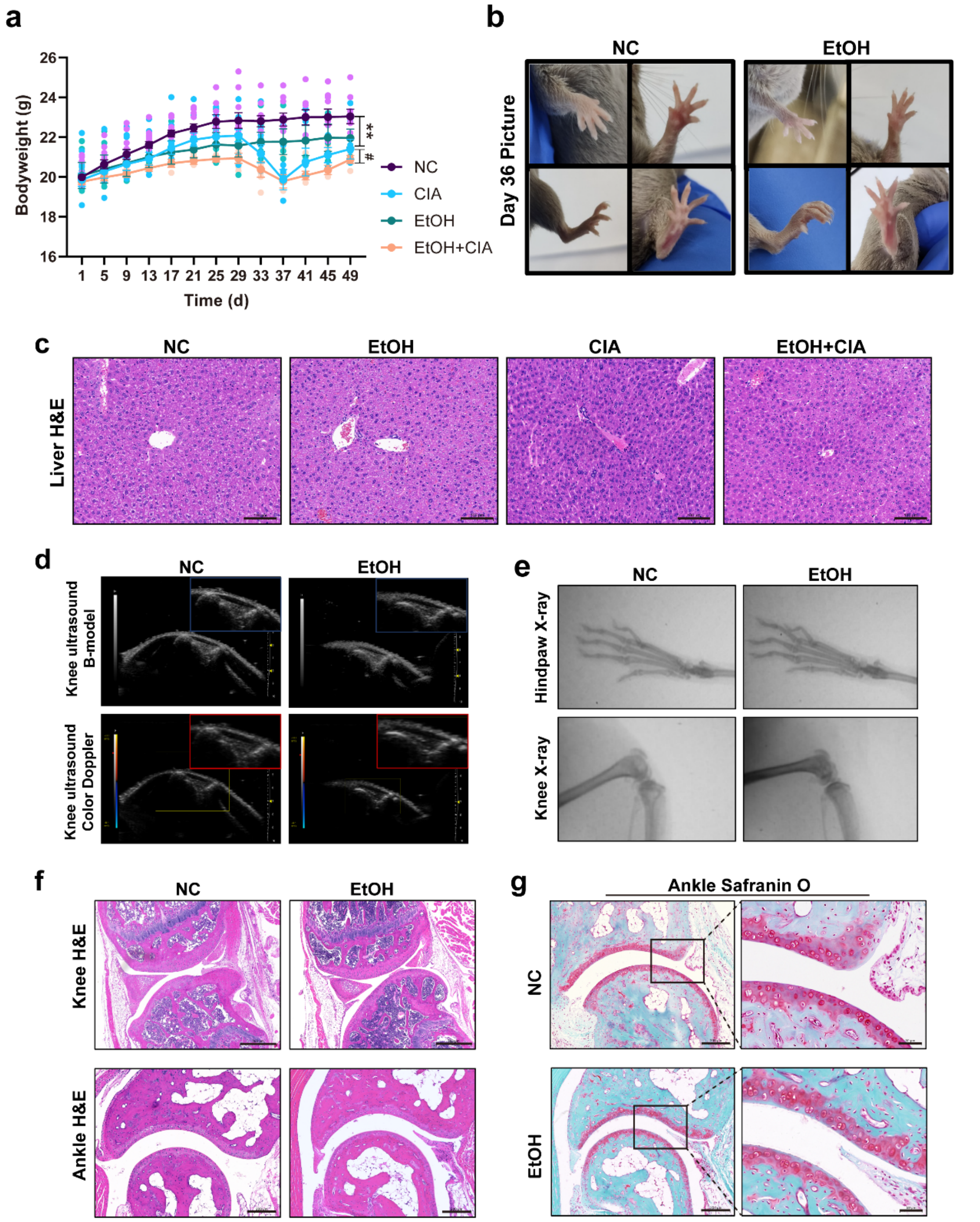

Supplementary Fig. 1 Low-dose ethanol consumption has no toxic effect on mice. **a** Dynamic change in body weight. The body weight changes of mice were recorded after the first immunization, and measured every three days. ** $p < 0.01$ versus the control group and # $p < 0.05$ versus the CIA group (NC n = 9, CIA n = 8, EtOH n = 5, EtOH+CIA n = 9). Data are described as mean ± SEM and compared with One-way ANOVA followed by Tukey's post hoc test. **b** Photographs of the hind paws and front paws on day 36 after the first immunization. **c** Liver H&E staining (Scale Bar: 100μm). **d-g** Imaging and histopathological staining of the feet of the normal control group and the ethanol consumption group. No abnormality is observed (Scale Bar: 200μm and 50μm).

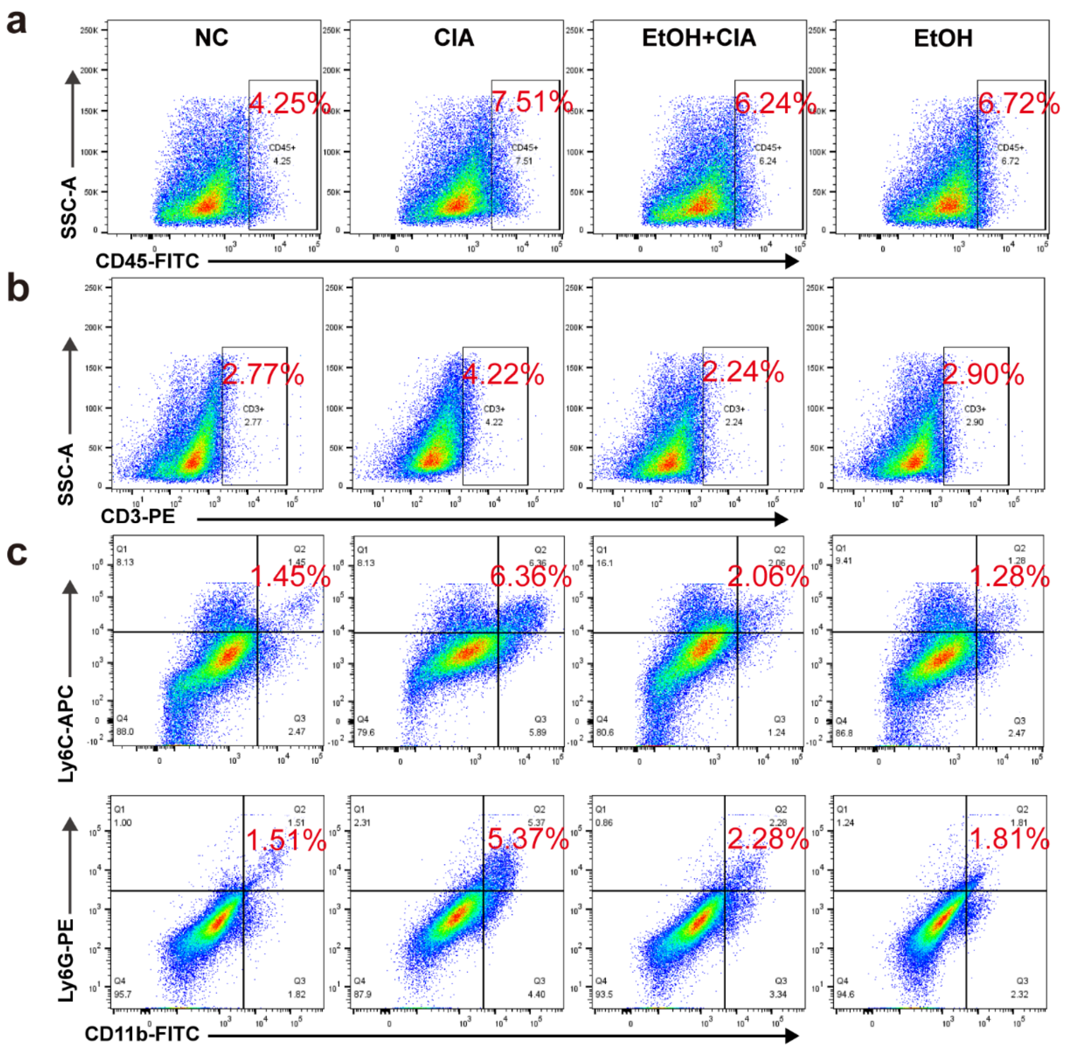

Supplementary Fig. 2 The proportion of immune cells in the paws of mice was detected by flow cytometry. a-c Quantitative analysis of white blood cells ($CD45^+$), T cells ($CD3^+$), monocytes and macrophages ($CD11b^+Ly6C^+$), and neutrophils ($CD11b^+Ly6G^+$) in mouse paw suspension by flow cytometry. Representative plots of cell populations in each group are presented.

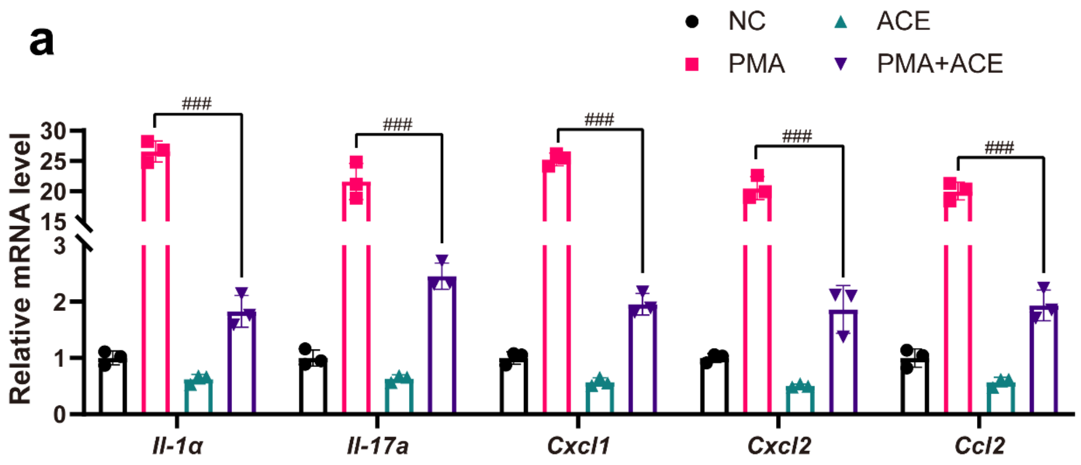

Supplementary Fig. 3 Acetate reduced the mRNA expression of inflammatory cytokines. a The mRNA expressions of inflammatory cytokine in dHL-60 cells by RT-qPCR. # $p < 0.05$, ## $p < 0.01$, and ### $p < 0.001$ represent significant differences between PMA group and PMA+ACE group (n = 3 per group). Data are described by mean ± SEM and analyzed with Two-way ANOVA followed by Sidak's post hoc test.

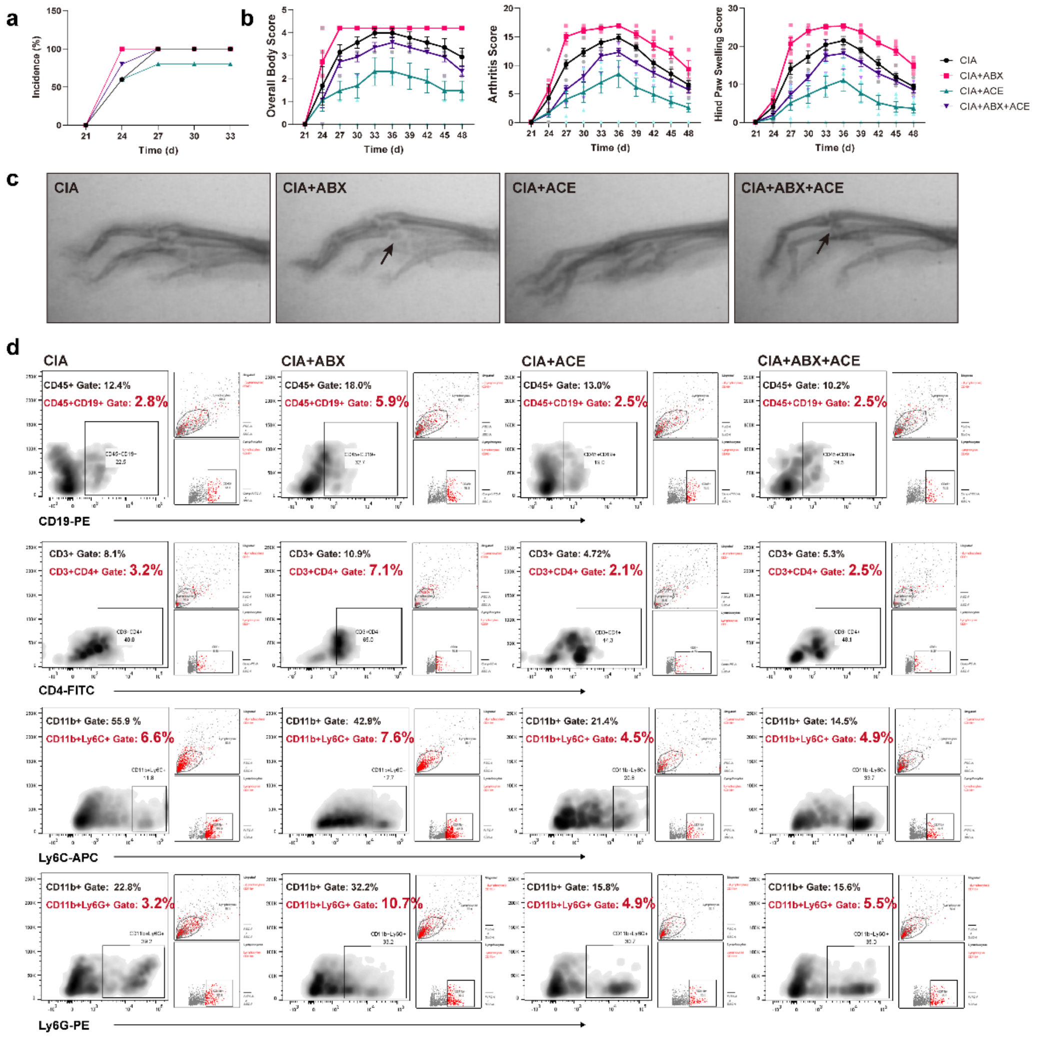

Supplementary Fig. 4 Exogenous acetate supplementation has a therapeutic effect on CIA mice. a Incidence of CIA, defined as Hind Paw Swelling Score > 0 per mouse. (n = 5 per group). **b** Arthritis severity during CIA, as assessed by arthritis score, including systemic score, paw swelling count, and arthritis index (n = 5 per group). Data are presented as mean ± SEM and analyzed with Two-way ANOVA followed by Sidak's post hoc test. **c** X-ray images of paws of mice show macroscopic evidence of arthritis. Swelling or scattered toe bones and markedly narrowed joint space (black arrow). **d** Quantitative analysis of B cells (CD45$^+$CD19$^+$), mature T lymphocytes (CD3$^+$CD4$^+$), monocytes and macrophages (CD11b$^+$Ly6C$^+$), and neutrophil (CD11b$^+$Ly6G$^+$). Representative plots of cell populations and gating strategies in each group are presented, and the percentage of labeled cells to total cells in each group is calculated.

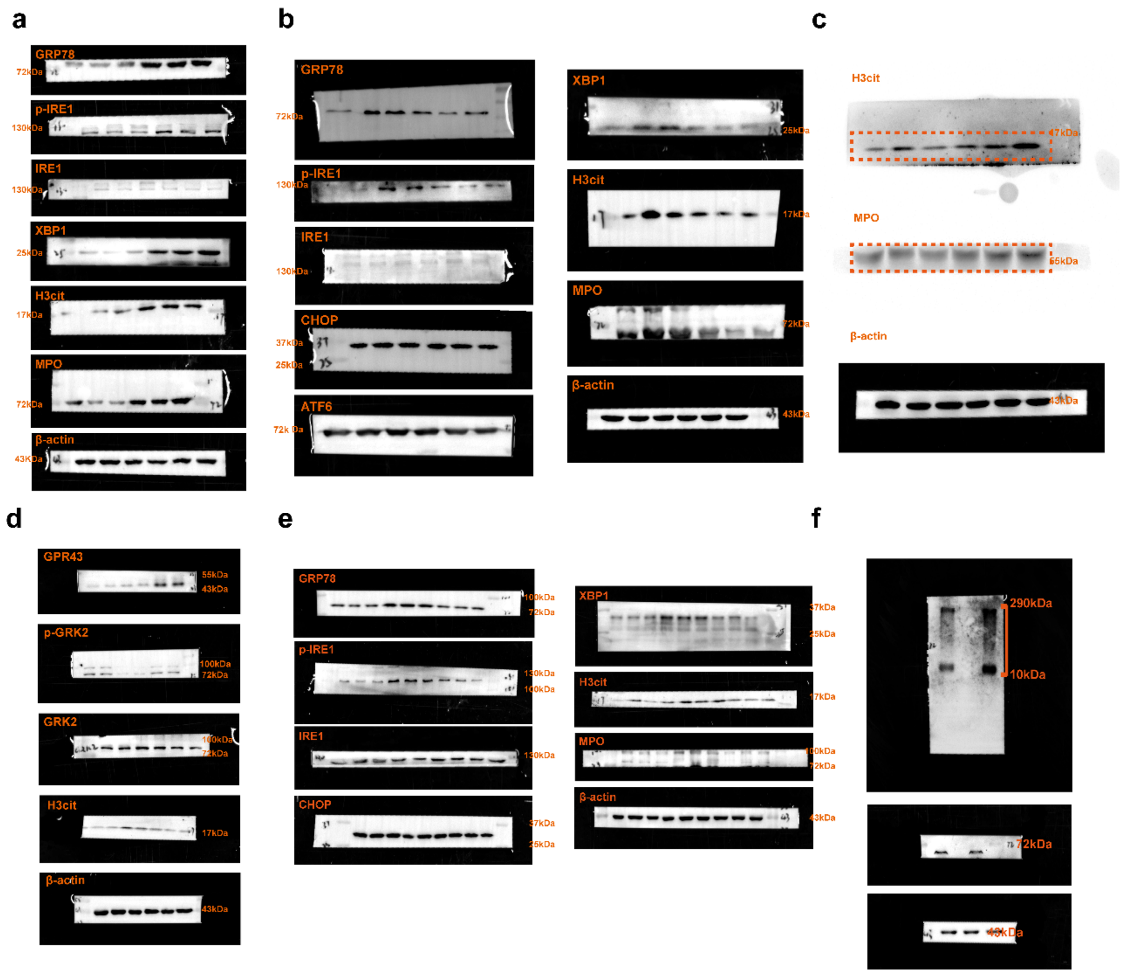

Supplementary Fig. 5 Western blots for Fig. 3 and Fig. 6. a Certain blots (Fig. 3c) were cut horizontally after Ponceau S staining to enable incubation in different primary antibodies, blots were also cut vertically to conserve antibody. The relative positions of the molecular weight markers (kDa) are shown. **b** Certain blots (Fig. 6d) were cut horizontally after Ponceau S staining to enable incubation in different primary antibodies, blots were also cut vertically to conserve antibody. The relative positions of the molecular weight markers (kDa) are shown. **c** The box highlights the region of immunoreactivity on each blot used in the main figure panels as indicated. Certain blots (Fig. 6e) were cut horizontally after Ponceau S staining to enable incubation in different primary antibodies, blots were also cut vertically to conserve antibody. The relative positions of the molecular weight markers (kDa) are shown. **d-f** Certain blots (Fig. 6f, g and h) were cut horizontally after Ponceau S staining to enable incubation in different primary antibodies, blots were also cut vertically to conserve antibody. The relative positions of the molecular weight markers (kDa) are shown.

2 Supplementary Videos

（The videos have been uploaded separately.）

Supplementary Video 1 Acetate reduces the formation of NETs. a Immunofluorescence staining of Sytox-green (green) in dHL-60 cells. The Formation of NETs is observed by Live cell Imaging System for 4 h after PMA treatment *in vitro*. **b** Immunofluorescence staining of Sytox-green (green) in dHL-60 cells. The Formation of NETs is observed by Live cell Imaging System for 4 h after acetate and PMA treatment *in vitro* (magnification, ×40).
