# Supplementary material for: Low-dose ethanol consumption inhibits neutrophil extracellular traps formation to alleviate rheumatoid arthritis
Source: Commun Biol. 2023 Oct 26;6:1088. doi: 10.1038/s42003-023-05473-y (PMC10603044; doi:10.1038/s42003-023-05473-y)
Supplement: Supplementary file 3 — Description of Additional Supplementary Data [file 42003_2023_5473_MOESM3_ESM.docx]

**Description of Additional Supplementary Files**

File name: Supplementary Video 1a

Description: Immunofluorescence staining of Sytox-green (green) in dHL-60 cells. The Formation of NETs is observed by Live cell Imaging System for 4 h after PMA treatment in vitro.

File name: Supplementary Video 1b

Description: Immunofluorescence staining of Sytox-green (green) in dHL-60 cells. The Formation of NETs is observed by Live cell Imaging System for 4 h after acetate and PMA treatment in vitro (magnification, ×40).
