# Supplementary material for: Low-dose ethanol consumption inhibits neutrophil extracellular traps formation to alleviate rheumatoid arthritis
Source: Commun Biol. 2023 Oct 26;6:1088. doi: 10.1038/s42003-023-05473-y (PMC10603044; doi:10.1038/s42003-023-05473-y)
Supplement: Supplementary file 6 — Reporting Summary [file 42003_2023_5473_MOESM6_ESM.pdf]

nature portfolio

Corresponding author(s): Ling-Ling Zhang

Last updated by author(s): 2023-10-12

Reporting Summary

Nature Portfolio wishes to improve the reproducibility of the work that we publish. This form provides structure for consistency and transparency in reporting. For further information on Nature Portfolio policies, see our Editorial Policies and the Editorial Policy Checklist.

Statistics

For all statistical analyses, confirm that the following items are present in the figure legend, table legend, main text, or Methods section.

n/a	Confirmed	
☐	☒	The exact sample size (*n*) for each experimental group/condition, given as a discrete number and unit of measurement
☐	☒	A statement on whether measurements were taken from distinct samples or whether the same sample was measured repeatedly
☐	☒	The statistical test(s) used AND whether they are one- or two-sided<br>*Only common tests should be described solely by name; describe more complex techniques in the Methods section.*
☐	☒	A description of all covariates tested
☐	☒	A description of any assumptions or corrections, such as tests of normality and adjustment for multiple comparisons
☐	☒	A full description of the statistical parameters including central tendency (e.g. means) or other basic estimates (e.g. regression coefficient) AND variation (e.g. standard deviation) or associated estimates of uncertainty (e.g. confidence intervals)
☒	☐	For null hypothesis testing, the test statistic (e.g. *F*, *t*, *r*) with confidence intervals, effect sizes, degrees of freedom and *P* value noted<br>*Give P values as exact values whenever suitable.*
☒	☐	For Bayesian analysis, information on the choice of priors and Markov chain Monte Carlo settings
☒	☐	For hierarchical and complex designs, identification of the appropriate level for tests and full reporting of outcomes
☒	☐	Estimates of effect sizes (e.g. Cohen's *d*, Pearson's *r*), indicating how they were calculated

Our web collection on statistics for biologists contains articles on many of the points above.

Software and code

Policy information about availability of computer code

Data collection	For date collection ：<br>Flow cvtometry: BD FACSuiteTM V.1.0.6 and FACSDivaTM V.8.0.2<br>RT-qPCR: QuantStudio 3 QuantStudioTM Design & Analysis Software V.1.4.1<br>TCS SP5 confocal microscope: Leica LAS X V.2.6.0<br>Pannoramic MIDI: 3D HISTECH V.1.4.0<br>Western blotting: lmageQuant LAS 4000 Control Software V.1.2.1.119<br>GC-MS: Thermo-Trace 1300 & Thermo-ISQ 7000<br>16S rRNA: Illumina NovaSeq PE250<br>X-rays: Leica, GTI-2000<br>Small animal ultrasonic instrument UBM: VEVO2100, Visualsonics
Data analysis	For data analysis:<br>Flow cytometry analysis: FlowJo V.10.5.3, BDTM Cytometric Bead Array FCAP Array Software V.3.0<br>maging analysis:lmageJ software V.1.52, QuPath V.0.4.3<br>Pannoramic MIDI analysis: SlideViewer V.2.5.0<br>GC-MS analysis: MassHunter Worksta software V.B.8.0<br>16S rRNA analysis: Qiime 2 V.QIIME2 2022.2<br>Confocal image analysis: Leica Application Suite X software<br>Statistics and analysis software: PRISM V.9.0.0

For manuscripts utilizing custom algorithms or software that are central to the research but not yet described in published literature, software must be made available to editors and reviewers. We strongly encourage code deposition in a community repository (e.g. GitHub). See the Nature Portfolio guidelines for submitting code & software for further information.

Data

Policy information about availability of data

All manuscripts must include a data availability statement. This statement should provide the following information, where applicable:

- Accession codes, unique identifiers, or web links for publicly available datasets
- A description of any restrictions on data availability
- For clinical datasets or third party data, please ensure that the statement adheres to our policy

All data that have been generated or analyzed during this study are included in the relevant databases and the associated supplementary files. The NCBI SRA accession number for the 16S rRNA data in this paper is PRJNA1026760 (https://www.ncbi.nlm.nih.gov/bioproject/PRJNA1026760). Supplementary videos (DOI: 10.6084/m9.figshare.22710202) and the source data for the graphs (DOI: 10.6084/m9.figshare.24286627) are available.

Research involving human participants, their data, or biological material

Policy information about studies with human participants or human data. See also policy information about sex, gender (identity/presentation), and sexual orientation and race, ethnicity and racism.

Reporting on sex and gender	In this study, synovial tissues of 8 patients (3 males/5 females) with RA were collected. During the experiment, all patients gave written informed consent. The information of RA patients was provided in Table 1. The remainder did not collect gender information on human participants. Sex and gender were not relevant to our hypotheses and only within-subject comparisons were made.
Reporting on race, ethnicity, or other socially relevant groupings	No information regarding race,ethnicity,and other socially relevant groupings was collected from human participants.These actors are not related to our hypothesis,and only within-subject comparisons were made.
Population characteristics	No demographic information was collected from human participants.These factors are not related to our hypothesis,and only within-subject comparisons were made.
Recruitment	For the collection of human synovial tissue for analysis of joint pathology,written informed consent was obtained from patients recruited from department of orthopedics, the Second People's Hospital of Hefei. For the collection volunteers blood,flyers were posted in the hospital,and participants called researchers to volunteer.
Ethics oversight	All experimental procedure were conducted in accordance with ethical regulation and use in China. The experimental protocol involving human subjects was approved by the Clinical Medical Research Ethics Committee of the First Affiliated Hospital of Anhui Medical University (approval number: 2022275).

Note that full information on the approval of the study protocol must also be provided in the manuscript.

Field-specific reporting

Please select the one below that is the best fit for your research. If you are not sure, read the appropriate sections before making your selection.

☒ Life sciences ☐ Behavioural & social sciences ☐ Ecological, evolutionary & environmental sciences

For a reference copy of the document with all sections, see nature.com/documents/nr-reporting-summary-flat.pdf

Life sciences study design

All studies must disclose on these points even when the disclosure is negative.

Sample size	Sample size is indicated in the figure legend for each experiments. For cell-based quantitative experiments, results of three independent biological replicates were used. For animal studies,we analyze a sufficient number of animals per group (minimum 5 animals) to evaluate differences between different groups. Statistical comparisons were performed using Student's t test for comparison between two groups or for paired comparisons, one-way ANOVA followed by Tukey post hoc test when more than two groups under same condition were involved, and two-way ANOVA followed by Sidak's or Tukey's post hoc test for comparison between two or more groups under two conditions. p-value less than 0.05 was considered significant.
Data exclusions	No data were excluded from the experiments.
Replication	Experimental findings were replicated using biological replicates within and between independent experiments. In almost all cases,findings were validated in at least two independent experiments,with the exception being experiments that were deemed too time-or cost-intensive (e.g., metabolomics, survival)and could be supported and extended by complimentary techniques (e.g., in vitro surrogate assay).
Randomization	Samples were randomly collected for each experimental group for analysis. Wherever possible, we collect all samples from each experimental condition and analyze them.
Blinding	Experimenters were blinded to score and grade of various indicators of mice; however, blinding was not always technically feasible. In these instances, samples were processed and/or positioned in an intercalated fashion to limit bias from group/batch effects. Subsequent data acquisition and analysis was performed in an automated fashion to reduce experimenter bias.

Reporting for specific materials, systems and methods

We require information from authors about some types of materials, experimental systems and methods used in many studies. Here, indicate whether each material, system or method listed is relevant to your study. If you are not sure if a list item applies to your research, read the appropriate section before selecting a response.

Materials & experimental systems

n/a	Involved in the study
☐	☒ Antibodies
☐	☒ Eukaryotic cell lines
☒	☐ Palaeontology and archaeology
☐	☒ Animals and other organisms
☒	☐ Clinical data
☒	☐ Dual use research of concern
☒	☐ Plants

Methods

n/a	Involved in the study
☒	☐ ChIP-seq
☐	☒ Flow cytometry
☒	☐ MRI-based neuroimaging

Antibodies

Antibodies used

For western blot: 1: 500 dilution for primary antibodies, 1: 20000 dilution for secondary antibodies For flow cytometry: 1: 200 dilution
For immunofluorescence assay: 1: 200 dilution Choice of antibodies were indicated in Methods section.
For commercially available antibodies, validation was performed by the manufacturer.
anti-GRP78 (western blotting): ER40402, HUABIO, lot: HJ1123. Validated WB in human cell lines on the manufacturer's website
anti-Phospho-IRE1(Ser 724) (western blotting): R26310, Zen BioScience, lot: KK0512.
anti-IRE1 (western blotting): 220399, Zen BioScience, lot: KK0510.
anti-CHOP (western blotting): ET1703-05, HUABIO, lot: HP0317.
anti-ATF6 (western blotting): R26445, Zen BioScience, lot: L19JL01.
anti-XBP1 (western blotting): R27438, Zen BioScience, lot: L19SE16.
anti-H3cit (western blotting): EPR17703, Abcam, lot: GR3250183-6.
anti-MPO (western blotting): 66177-1-lg90, Proteintech, lot: 10025998.
anti-GPR43 (western blotting): DF2746, Affinity Biosciences, lot: 81z6719.
anti-GRK2 (western blotting): WL03072, Wanlei BioScience, lot: GO4283072.
anti-p-GRK2 (western blotting): AF3697, Affinity Biosciences, lot: 36p7755.
anti-Ubiquitin (western blotting): 382766, Zen BioScience, lot: L10N004.
anti-β-Actin (western blotting): AF7018, Affinity Biosciences, lot: 12w2944.
anti-H3cit (Immunofluorescence assay): EPR20358-120, Abcam, lot: 1000318-1.
anti-MPO(Immunofluorescence assay): R25062, Zen BioScience, lot: L04AU25.
anti-Phospho-IRE1(Ser 724) (Immunofluorescence assay): R26310, Zen BioScience, lot: KK0512.
Goat anti-Mouse IgG (H+L) Secondary Antibody, anti-Alexa Fluor 647 (Immunofluorescence assay): S0014, Affinity Biosciences, lot: 6019420.
Goat anti-Rabbit IgG (H+L) Secondary Antibody, anti-Alexa Fluor 488 (Immunofluorescence assay): E-AB-1055, Elabscience, lot: SY0155.
anti-CD45-FITC (Flow cytometric analysis): 103107, BioLegend, lot: B350443.
anti-CD19-PE (Flow cytometric analysis): 30227, BioLegend, lot: B188905.
anti-CD3-PE (Flow cytometric analysis): 100308, BD Pharmingen, lot: B290417.
anti-CD4-FITC (Flow cytometric analysis): 100519, BD Pharmingen, lot: B350912.
anti-CD11b-FITC (Flow cytometric analysis): 101206, BioLegend, lot: B349919.
anti-Ly6G-PE (Flow cytometric analysis): 127607, BioLegend, lot: B332036.
anti-Ly6C- APC (Flow cytometric analysis): 128015, BioLegend, lot: B322022.

Validation

Antibodies were validated by their respective manufacturers to ensure specific binding activity and lot-to-lot consistency.Additional details may be found on the manufacturers'website by searching for the respective catalog number listed above. This information was used to further validate the specificity.

Eukaryotic cell lines

Policy information about cell lines and Sex and Gender in Research

Cell line source(s)

The following cell lines were used in this work:
The HL-60 cells (Procell Life Science & Technology Co.,Ltd, China) were cultured in IMDM medium, supplemented with 20% fetal bovine serum and 1% penicillin-streptomycin solution, in a 37°C, 5% CO2 cell incubator. 1.3% DMSO was added to the culture medium for 7 days to induce HL-60 cells into neutrophil-like differentiation of HL-60 (dHL-60) cells to study the biological function of neutrophils.

Authentication

HL-60 cells were authenticated by STR; Primary neutrophils confirmed to express cell lineage marker by Flow Cytometry for authentication. After incubation with CD15-PE antibody, flow cytometry was used to identify whether the inducted dHL-60 cells was successful.

Mycoplasma contamination

All cell lines used in this work have been tested monthly to ensure negative for mycoplasma contamination.

Commonly misidentified lines
(See ICLAC register)

No commonly misidentified cell lines used.

Animals and other research organisms

Policy information about studies involving animals; ARRIVE guidelines recommended for reporting animal research, and Sex and Gender in Research

Laboratory animals	DBA/1 mice were purchased from Gem Pharmatech (Nanjing, China). 7–8-week-old male DBA/1 mice were used for experiments, which is the week age usually used in most RA modeling experiments. All mice were maintained under specific pathogen-free conditions at 25[illegible]with 12 h light and dark cycles in accordance with current ethical regulations for animal care and use in China. In the experiment, caging bias was taken into account. Instead of feeding them in an independent ventilated cage, they were all placed in the same environment open to the outside.
Wild animals	This study did not involve wild animals.
Reporting on sex	It is reported that no gender difference in CIA mice (Brand et al., 2007). In order to avoid the impact of various hormone fluctuations, such as estrogen, in the female menstrual cycle on the pathogenesis of RA, only male mice are used as experimental subjects (Miyoshi et al.,2018).
Field-collected samples	The study did not involve samples collected from the field.
Ethics oversight	This study was approved by the Experimental Animal Ethics Committee of Anhui Medical University (approval number: 20220454). Animal welfare and experimental procedures were strictly in accordance with the guidelines for the care and use of laboratory animals.

Note that full information on the approval of the study protocol must also be provided in the manuscript.

Plants

Seed stocks	*Report on the source of all seed stocks or other plant material used. If applicable, state the seed stock centre and catalogue number. If plant specimens were collected from the field, describe the collection location, date and sampling procedures.*
Novel plant genotypes	*Describe the methods by which all novel plant genotypes were produced. This includes those generated by transgenic approaches, gene editing, chemical/radiation-based mutagenesis and hybridization. For transgenic lines, describe the transformation method, the number of independent lines analyzed and the generation upon which experiments were performed. For gene-edited lines, describe the editor used, the endogenous sequence targeted for editing, the targeting guide RNA sequence (if applicable) and how the editor was applied.*
Authentication	*Describe any authentication procedures for each seed stock used or novel genotype generated. Describe any experiments used to assess the effect of a mutation and, where applicable, how potential secondary effects (e.g. second site T-DNA insertions, mosiacism, off-target gene editing) were examined.*

Flow Cytometry

Plots

Confirm that:

- [x] The axis labels state the marker and fluorochrome used (e.g. CD4-FITC).
- [x] The axis scales are clearly visible. Include numbers along axes only for bottom left plot of group (a 'group' is an analysis of identical markers).
- [x] All plots are contour plots with outliers or pseudocolor plots.
- [x] A numerical value for number of cells or percentage (with statistics) is provided.

Methodology

Sample preparation	The mouse feet were cut into tissue blocks of 3-4 mm in a 1.5 mL EP tube, and added Hank's equilibrium salt solution (1 mL), collagenase type II (1 g/mL, 2 µL) and CaCl2 solution (3 mM, 5 µL). After incubated at 37[illegible]for 4 h, taken the supernatant by centrifugation, washed with PBS 3 times after passing through a nylon sieve to obtain dispersed cells. Fluorescent antibodies were added to the treated single cell suspension (1×10^6/100 µL), and incubated at 4°C in the dark for 30 min. Then, after centrifugation at 300 g, PBS was added and the cells were repeatedly washed 3 times. Finally, 300 µL PBS was added to resuspend, and then the results of cell sorting were observed.
Instrument	Flow cytometry acquisition was performed using an BD FACSCanto VerseII (BD Bioscience).
Software	Acquisition was accomplished with BD FACSuiteTM V.1.0.6 and FACSDivaTM v8.0.2,and analysis was performed with Treestar FlowJo V.10.8.1.
Cell population abundance	Approximately 100,000-300,000 events of interest were captured per sample. For MACS sorted cells, the purity of relevant population was validated by FACS analysis.
Gating strategy	Cells were gated for lymphocyte population (FSC-A/SSC-A), then singlets were gated in FSC-W/FSC-H and SSC-W/SSC-H and live cells were selected by Live/Dead Fixable Dye. We designed the experiment with reference to the work of Shin AE. et al. [illegible]

(Gastroenterology. 2023) and Huang et al.(Hepatology. 2022), B cells (CD45+CD19+), mature T lymphocytes (CD3+CD4+), monocyte-macrophages (CD11b+Ly6C+), and neutrophil (CD11b+Ly6G+).

☒ Tick this box to confirm that a figure exemplifying the gating strategy is provided in the Supplementary Information.
